# Terbutaline, forskolin and cAMP reduce secretion of aqueous humour in the isolated bovine eye

**DOI:** 10.1371/journal.pone.0244253

**Published:** 2020-12-21

**Authors:** Mohammad Shahidullah, William Stuart Wilson, Kazi Rafiq, Mahmudul Hasan Sikder, Jannatul Ferdous, Nicholas Anthony Delamere

**Affiliations:** 1 Department of Physiology and the Department of Ophthalmology and Vision Science, University of Arizona, Tucson, AZ, United States of America; 2 College of Medical, Veterinary and Life Sciences, University of Glasgow, Glasgow, United Kingdom; 3 Department of Pharmacology, Bangladesh Agricultural University, Mymensing, Bangladesh; Cinvestav-IPN, MEXICO

## Abstract

In order to elucidate involvement of cyclic AMP and intracellular Ca^2+^,[Ca^2+^]_i_, in the modulation of aqueous humour formation (AHF), we studied the effects of terbutaline, forskolin and 8-Br-cAMP in the isolated bovine eye. We also studied the interaction of cAMP on calcium signaling in cultured ciliary epithelial (CE) cells. Drug effects on AHF were measured by fluorescein dilution. Drug effects on [Ca^2+^]_i_ were studied by the fura-2 fluorescence ratio technique. Terbutaline (100 nmol-100 M), forskolin (30 nM-100 M) or 8-Br-cAMP (100 nM– 10 μM), administered in the arterial perfusate produced significant reductions in AHF. The AH reducing effect of terbutaline was blocked by a selective inhibitor of protein kinase A (KT-5720). ATP (100 M) caused a rapid, transient (peak) increase in [Ca^2+^]_i_ followed by a sustained plateau phase lasting more than 5 minutes. Preincubation of the cells (6 min) with terbutaline, forskolin or 8-Br-cAMP significantly reduced the peak calcium response to ATP. The sustained plateau phase of the response, on the other hand, was augmented by each of the agents. KT-5720 partially reversed the inhibitory effect of terbutaline on the peak and totally inhibited its effect on the plateau phase. These data indicate: (a) that AHF in the bovine eye can be manipulated through cyclic AMP, operating via protein kinase A, (b) that protein kinase A can affect [Ca^2+^]_i_ homeostasis, (c) that calcium release from the intracellular store, not the entry, affects AHF, and (d) that interaction of [Ca^2+^]_i_ with cAMP plays a role in modulating AH secretion.

## Introduction

Aqueous humour (AH) is secreted into the posterior chamber of the eye by the ciliary epithelium (CE), a double layer of cells located on the surface of the muscular ciliary body. Cyclic AMP (cAMP) has long been considered to be an important regulator of aqueous humour formation (AHF) and this area has been extensively studied [1–4]. However, the mechanisms involved are not clearly understood, not least, since reduction of intraocular pressure (IOP) and AHF by both β-adrenergic agonists and antagonists occurs in some species [5–11]. In the bovine isolated perfused eye we have reported that AHF is suppressed by the β_2_ agonist, terbutaline, but that there seems to be no correlation between drug effects on AHF and those on the cAMP content of the CE [12].

In many tissues, other important intracellular second messengers have become firmly established including Ca^2+^. Ca^2+^ is well established as a regulator of a number of cellular functions including secretion [13,14]. Many workers have reported “transients” or sudden changes in intracellular calcium, [Ca^2+^]_i_, in cultured CE cells [15–20]. We have previously identified and characterised P2Y2 receptors in cultured bovine CE [18]. Stimulation of these receptors with ATP or UTP causes [Ca^2+^]_i_ release and a rise in intracellular free Ca^2+^ concentration [18]. Release of Ca^2+^ has also been reported in CE cells by stimulation of various other receptors including histamine H1 and vasopressin V1 receptors [21–23], 1 and 2-adrenergic, muscarinic and adenosine receptors [15–17,19].

We have previously published evidence of links between AH production and [Ca^2+^]_i_ by examining the effects of drugs acting through cyclic GMP on AHF in bovine perfused eye and on ATP-induced increase in [Ca^2+^]_i_ in bovine cultured CE [24]. Such data help to correlate physiological effects with biochemical phenomena. In the present investigation, we present data establishing similar links using drugs which act via cAMP and which affect both AHF and Ca^2+^ signaling in bovine CE cells.

## Materials and methods

### Drugs and chemicals

ATP, terbutaline, forskolin, 8-bromo cAMP, bovine serum albumin (fraction V), EDTA, EGTA, DMSO, acetoxymethyl ester of fura-2 (fura-2 AM), ionomycin, HEPES and gentamycin were all purchased from Sigma Chemical Co. KT-5720 was obtained from Calbiochem. Dulbecco’s modified Eagle’s medium (DMEM), foetal bovine serum (FBS), new-born calf serum (NCS) and trypsin EDTA (1X) were obtained from Life Technologies and collagenase A from Boehringer Mannheim. MnCl2 was obtained from BDH and NiCl_2_ from Hopkin and Williams. All other reagents were of analytical grade. Drugs were dissolved in appropriate concentrations either in distilled water or in dimethyl sulfoxide (DMSO) according to their solubility.

### Bovine eye

Bovine eyes were used for whole eye perfusion study to estimate aqueous humor secretion and for obtaining primary culture of ciliary epithelium to measure intracellular calcium. Bobine eyes were obtained from a local abattoir. The use of animal tissue was approved by the University of Arizona, Institutional Animal Care and Use Committees (Protocol number: 12–395) and conformed to the ARVO Resolution for the Use of Animals in Ophthalmic and Vision Research.

### Estimation of AHF

AHF rate was measured by the fluorescein dilution technique as described earlier [25,26]. In brief, bovine eyes were perfused through the ophthalmic artery at 37C with an oxygenated Krebs' solution comprising (mM): NaCl, 118; KCl, 4.7; CaCl2, 2.5; KH2PO4, 1.2; MgSO4, 1.2; NaHCO3, 25; glucose, 11.5; ascorbate, 0.05. Drugs were administered at fixed concentrations in the arterial perfusate. AHF rate was estimated for 30 min before addition of drug solution into the perfusate to obtain control values (this excluded individual variation among different eyes). Estimation of AHF rate was then restarted 15–20 min after exposure to perfusate containing the drug and continued for a further 60 min.

### Culture of CE cells and measurement of intracellular Ca^2+^

The tips (1–2 mm) of ciliary processes were isolated from bovine eyes and CE cells were cultured as previously described [12]. Cells from the 3^rd^ or 4^th^ passage were placed on sterile glass coverslips and cultured overnight before being loaded with fura-2 for studies on [Ca^2+^]_i_. The cells were incubated for 30 min at 37C with fura-2 AM (2 M) in HEPES (25 mM)-buffered DMEM containing bovine serum albumin (1%) under constant gentle shaking. The coverslips containing cells loaded with fura-2 were transferred to HEPES (10 mM)-buffered Krebs' solution (pH 7.4) containing (mM): NaCl, 118; KCl, 4.8; MgSO4, 1.0; NaHCO3, 2.4; glucose, 11.0; HEPES 10.0 and CaCl2, 1.8 and left at room temperature for 20 min, to allow for hydrolysis of fura-2 AM to the Ca2+-sensitive acid form. Coverslips with fura-2 loaded cells were then mounted in a small bath attached to the stage of an inverted microscope, where the cells were continuously superperfused (approximately 2.5 ml/min, at 37°C) with oxygenated HEPES buffered Krebs’ solution mentioned above. All experiment was carried out using this HEPES-buffered Krebs’ solution, but in some, the CaCl_2_ was omitted, as indicated in the result. In experiments involving NiCl_2_ (4mM), the same solution was used, except that MgSO_4_ was replaced with 1.0 mM MgCl_2_ and NaHCO_3_ was omitted. The drugs were added to the perfusate at appropriate concentration.

Measurement and estimation of [Ca^2+^]_i_ was done according to the dual excitation method of Grynkiewicz [27] and using Quanticell 500 imaging system (Applied Imaging). The Fura-2 fluorescence signal (Ex 340/380 nm; Em 515nm) were acquired with a Nikon Diaphot inverted microscope and a CCD camera. At the end of each experimental run, background auto fluorescence was obtained according to Hallam et al [28]. Ionomycin (2 μM) was added to permeabilize the cells to divalent cations and MnCl_2_ (2mM) was added to quench fura-2 fluorescence. After subtraction of autofluorescence, the corrected fluorescence values obtained, following excitation at 340 nm, were divided by those obtained at 380 nm, giving a corrected ratio (R). [Ca^2+^]_i_ was then computed using the Grynkiewicz equation,

$${\left[{\text{Ca}}^{2+}\right]}_{\text{I}}={\text{K}}_{\text{d}}\times \frac{\left(\text{R}–{\text{R}}_{\text{min}}\right){\text{S}}_{\text{f}2}}{\left({\text{R}}_{\text{max}}-\text{R}\right){\text{S}}_{\text{b}2}}$$

and a resultant experimental trace of [Ca^2+^]_i_ versus time obtained. R_max_ and R_min_ are the maximal and minimal fluorescence ratios of fura-2 obtained in a saturating concentration of calcium and in calcium-free medium (with 40 mM EGTA), respectively. S_f2_ and S_b2_ are the fluorescence values, obtained at 380 nm in the absence of calcium and in the presence of saturating levels of calcium, respectively. The K_d_ for the fura-2-Ca^2+^ complex was assumed to be 224 nM at 37°C [27]. Values of Rmax, Rmin, Sf2 and Sb2 were experimentally determined in fura-2 loaded CE cell suspensions according to the method described earlier [18]. The following calibration values were obtained and used throughout to calculate [Ca^2+^]_i_: Rmax, 17.59 0.34 (n = 4); Rmin, 0.91 0.04 (n = 4); Sf2/Sb2, 6.89 0.02 (n = 13).

### Statistical analysis

Data on AHF were subjected to one-way analysis of variance (ANOVA) and paired Student’s t-test. Data on [Ca^2+^]_i_ were analysed using one-way ANOVA followed by Bonferoni’s multiple comparison test. Graphs were drawn using the GraphPad Prism computer package.

## 3. Results

### Effects of terbutaline and forskolin and 8-Br-cAMP on AH formation

**Fig 1:**
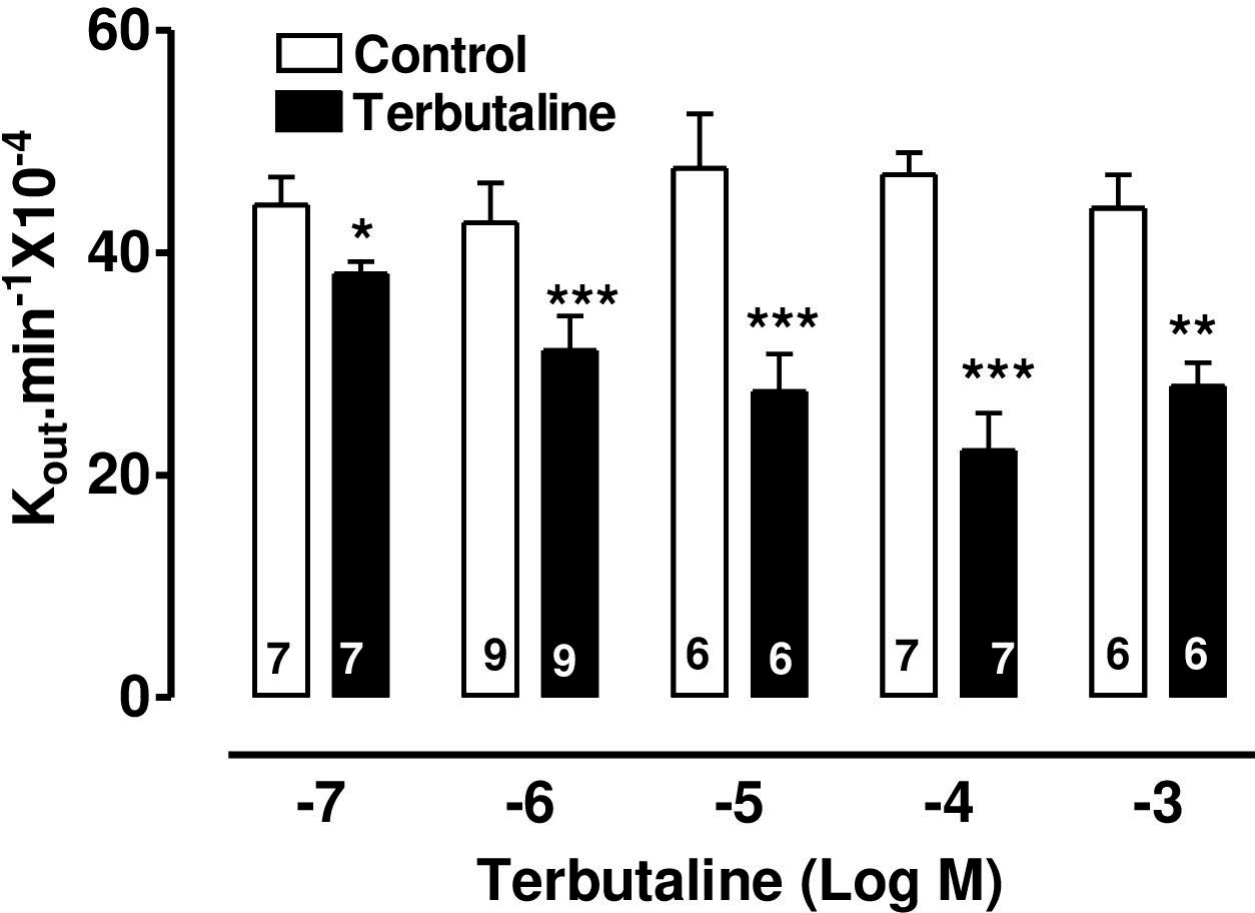
The effect of terbutaline on AH formation rate in the bovine isolated perfused eye measured by fluorescein dilution technique. AH formation rate was expressed as the rate constant (K_out_.min^-1^ × 10^−4^), defined as the slope of the regression line drawn on LN (Log_e_) of the changes of fluorescence with time (min). Each value is a mean s.e.mean of the number (n) of experiments shown at the base of each column. In order to eliminate individual variation, control and treated data were obtained from the same eye. AH formation rate measured during the first 30 min prior to drug application was taken as the control value. AH formation rate for the subsequent 40–60 min after establishment of drug effects was taken as the treated value. Note that after addition of drug, 20 min stabilization period was allowed to establish drug effect. Significance of differences from controls; * p < 0.05, ** p < 0.01 and *** p < 0.001.

**Fig 2:**
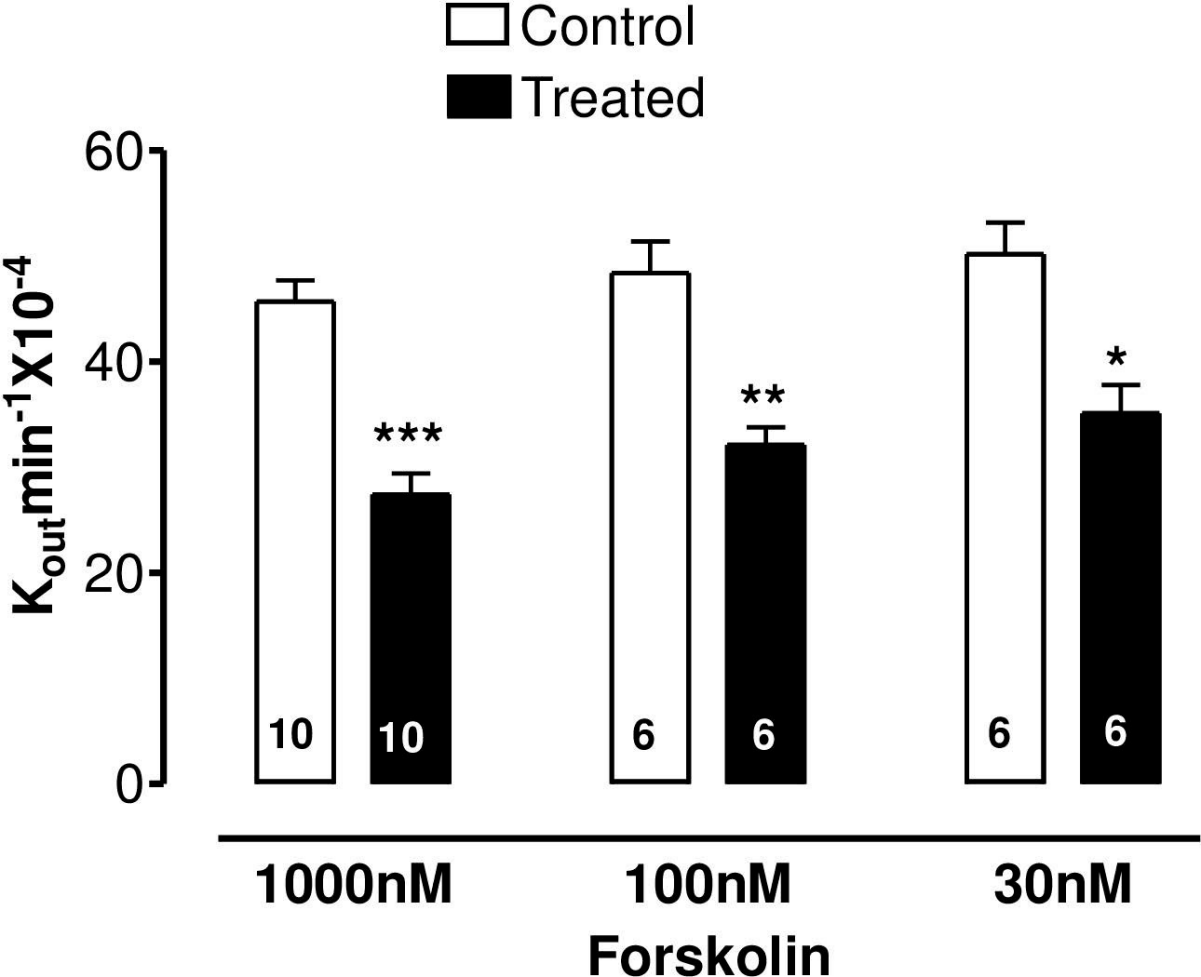
The effect of forskolin on AH formation rate in the bovine isolated perfused eye measured by fluorescein dilution technique. AH formation rate was expressed as the rate constant (K_out_.min^-1^ × 10^−4^), defined as the slope of the regression line drawn on LN (Log_e_) of the changes of fluorescence with time (min). Each value is a mean s.e.mean of the number (n) of experiments shown at the base of each column. In order to eliminate individual variation, control and treated data were obtained from the same eye. AH formation rate measured during the first 30 min prior to drug application was taken as the control value. AH formation rate for the subsequent 40–60 min after establishment of drug effects was taken as the treated value. Note that after addition of drug, 20 min stabilization period was allowed to establish drug effect. Significance of differences from controls; * p < 0.05, ** p < 0.01 and *** p < 0.001.

**Fig 3:**
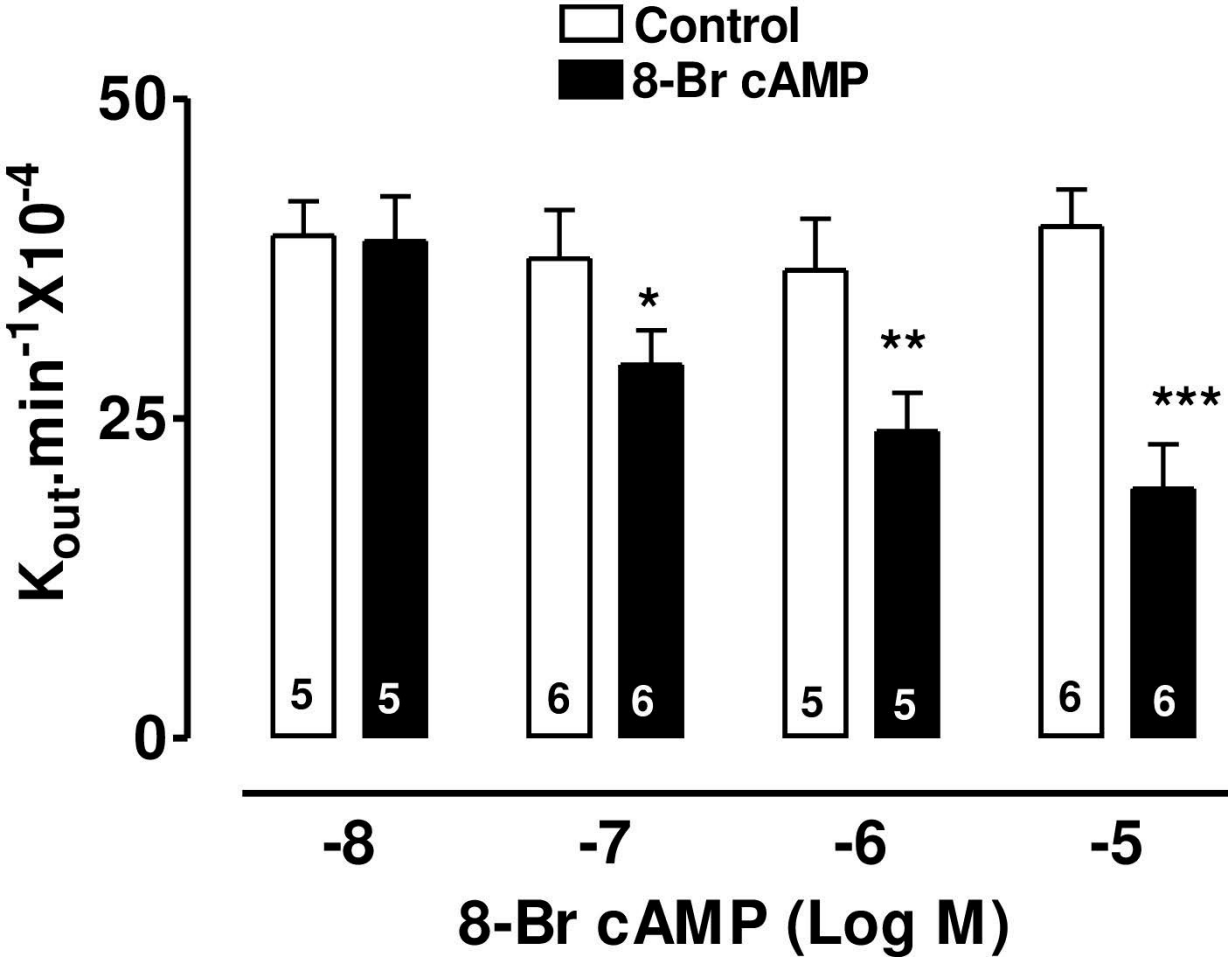
The effect of 8-Br-cAMP on AH formation rate in the bovine isolated perfused eye measured by fluorescein dilution technique. AH formation rate was expressed as the rate constant (K_out_.min^-1^ × 10^−4^), defined as the slope of the regression line drawn on LN (Log_e_) of the changes of fluorescence with time (min). Each value is a mean s.e.mean of the number (n) of experiments shown at the base of each column. In order to eliminate individual variation, control and treated data were obtained from the same eye. AH formation rate measured during the first 30 min prior to drug application was taken as the control value. AH formation rate for the subsequent 40–60 min after establishment of drug effects was taken as the treated value. Note that after addition of drug, 20 min stabilization period was allowed to establish drug effect. Significance of differences from controls; * p < 0.05, ** p < 0.01 and *** p < 0.001.

The specific β_2_-adrenoceptor agonist, terbutaline, caused a highly significant reduction of AH formation rate (Fig 1) as did the direct stimulator of adenylate cyclase, forskolin, (Fig 2) and 8-Br-cAMP, the cell permeable analogue of endogenous cAMP (Fig 3).

### Effects KT-5720 on the AH-reducing action terbutaline

**Fig 4:**
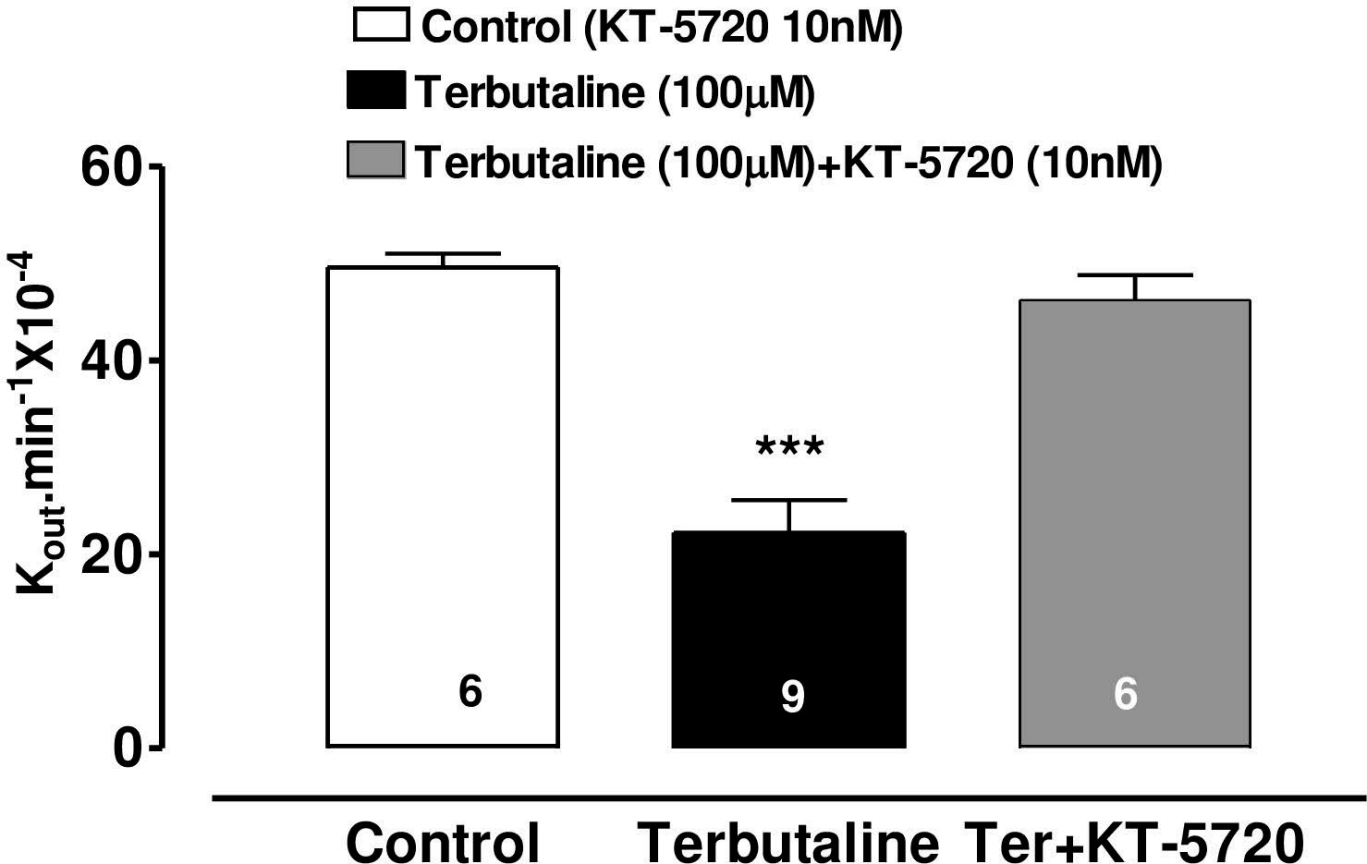
Effect of KT-5720 (10nM) on the inhibitory effect of terbutaline (100μM) on AH formation in the bovine perfused eye. Stock solution of the PKA inhibitor (KT-5720) in DMSO was added to the perfusate to obtain a final concentration of 10 nM. AH formation rate (shown as K_out_.min^-1^ 10^−4^) was measured in separate groups of eyes for 90 min after injection of vehicle or drug. Each value is a mean s.e.mean of the number (n) of experiments shown at the base of each column. Control: injection of vehicle and PK inhibitor. Significance of difference from control; *** p < 0.001.

KT-5720 (10nM), a specific inhibitor of cAMP-dependent protein kinase (PKA), was added to the perfusate 10 minutes prior to terbutaline and this abolished the AH-suppressing effect of terbutaline (100 M) (Fig 4).

### Effects of terbutaline, forskolin and 8-bromo cAMP on ATP-induced Ca^2+^ transients in CE

**Fig 5:**
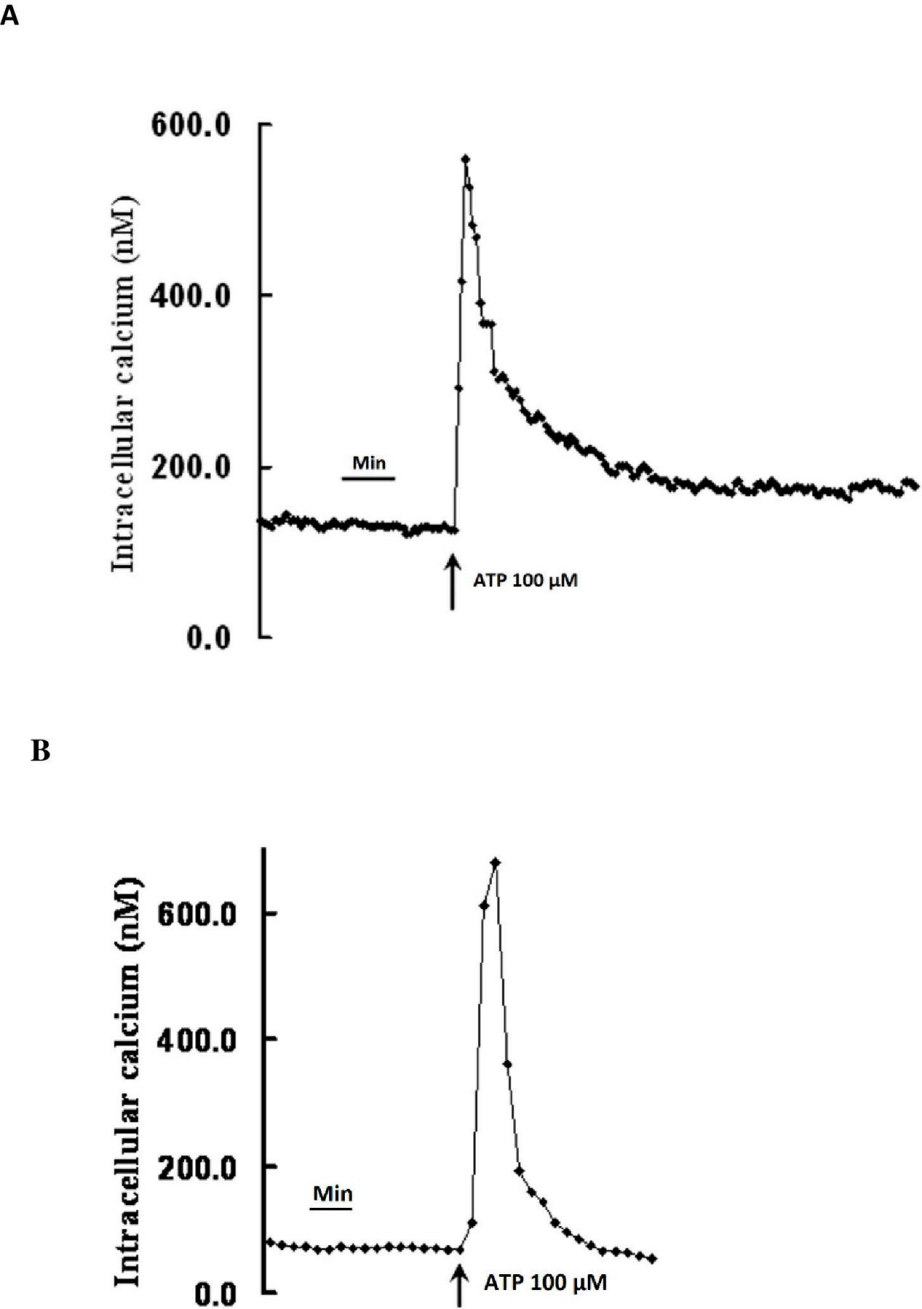
Increase in intracellular Ca^2+^ in bovine cultured ciliary epithelial cells in response to ATP (100 μM). (A) Typical trace, obtained in the presence of 1.8mM extracellular calcium, shows a rapid increase in [Ca^2+i^]_i_ which is followed by a rapid decline and then a prolonged plateau above baseline. (B) Typical trace obtained in the absence extracellular calcium shows a rapid increase in [Ca^2+i^]_i_ which is followed by a rapid decline to baseline without forming the plateau.

Addition of 100 M ATP to cultured CE, in the presence of extracellular calcium, caused a highly reproducible Ca2+ transient whose peak was followed by a slow decline and plateau (see typical trace in Fig 5A). The plateau phase was completely absent when cells were exposed to ATP in calcium free solution (Fig 5B), indicating that the plateau phase is due to entry of calcium from extracellular space. The peak of the Ca2+ transient was usually reached within 30s.

**Fig 6:**
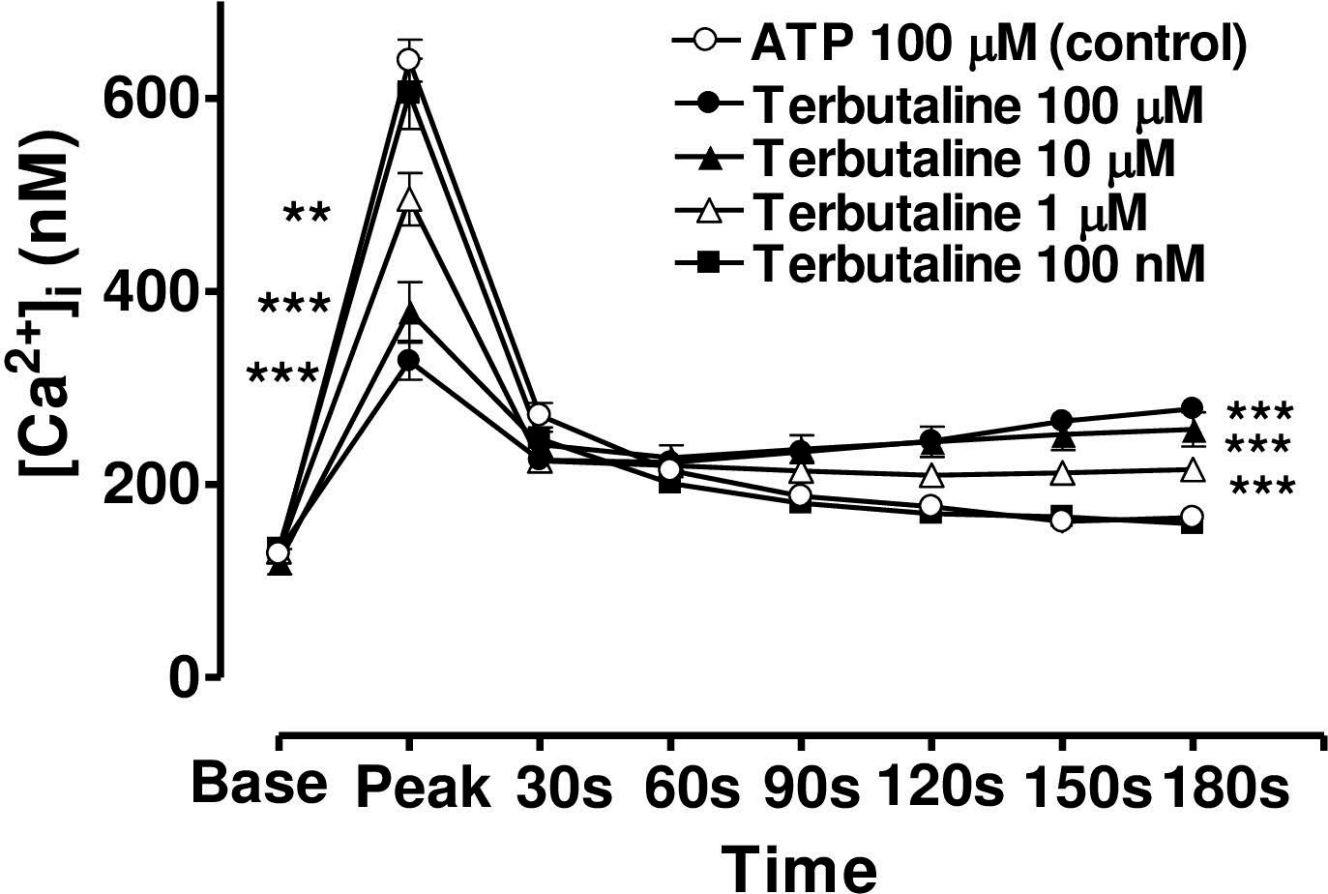
Effect of increasing concentration of terbutaline on the Ca^2+^-mobilizing response of ATP (100μM) in bovine cultured CE, both at the peak of the Ca^2+^ transient and at selected time intervals thereafter. Cultured cells were incubated for 6 min with appropriate concentration of terbutaline in HEPES bufererd Krebs’ solution containing 1.8mM calcium. The cells were then exposed to 100μM of ATP. Each point represents a mean±s.e.mean of 8–10 experiments. Significance of differences between incubations with or without terbutaline; ** p < 0.01 and *** p < 0.001, Bonferroni’s multiple comparison test. Base value indicates the [Ca^2+^]_i_ prior to addition of ATP.

**Fig 7:**
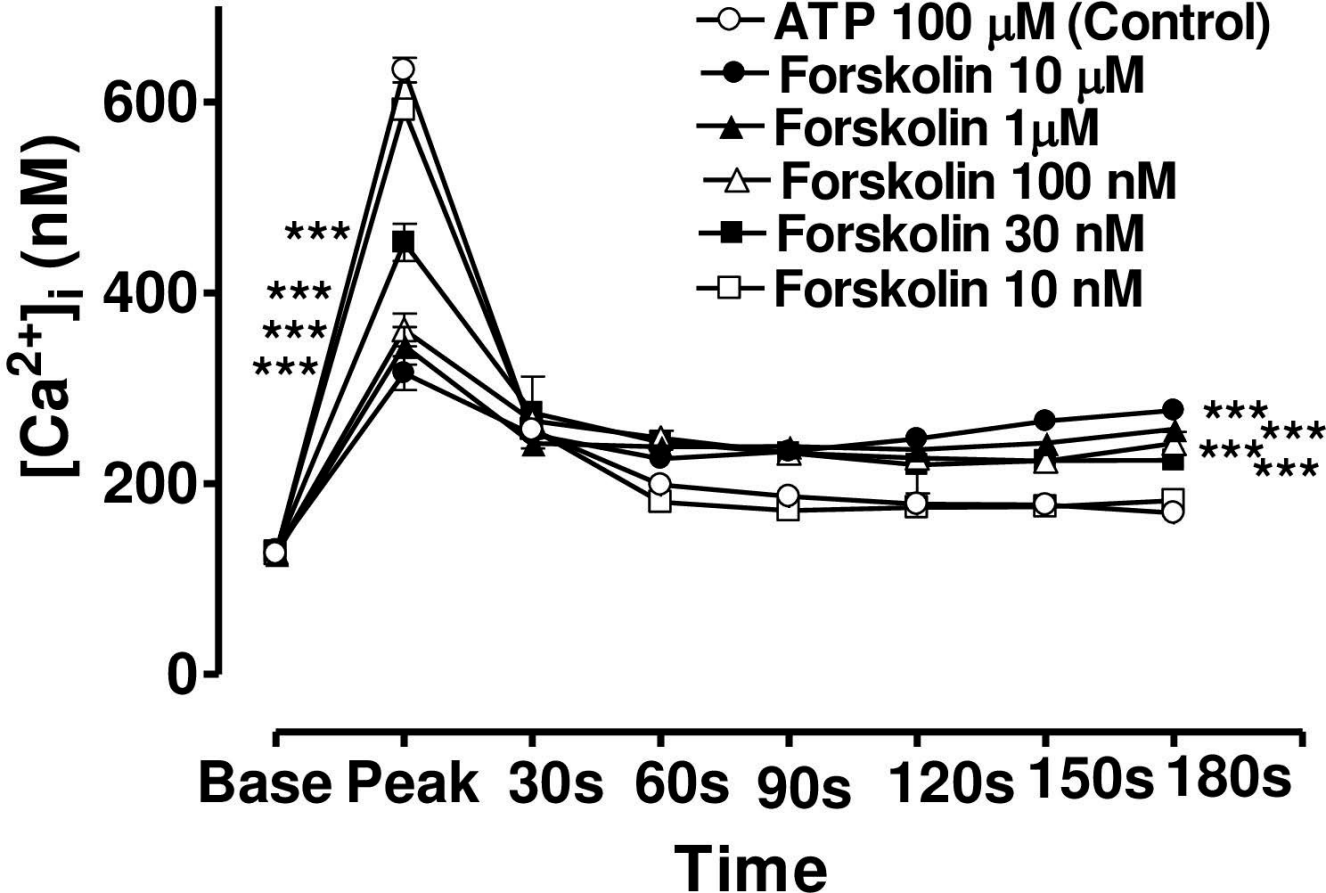
Effect of increasing concentration of forskolin on the Ca^2+^-mobilizing response of ATP (100μM) in bovine cultured CE, both at the peak of the Ca^2+^ transient and at selected time intervals thereafter. Cultured cells were incubated for 6 min with appropriate concentration of forskolin in HEPES buffered Krebs’ solution containing 1.8mM calcium. The cells were then exposed to 100μM of ATP. Each point represents a mean±s.e.mean of 8–15 experiments. Significance of differences between incubations with or without forskolin; ** p < 0.01 and *** p < 0.001, Bonferroni’s multiple comparison test. Base value indicates the [Ca^2+^]_i_ prior to addition of ATP.

**Fig 8:**
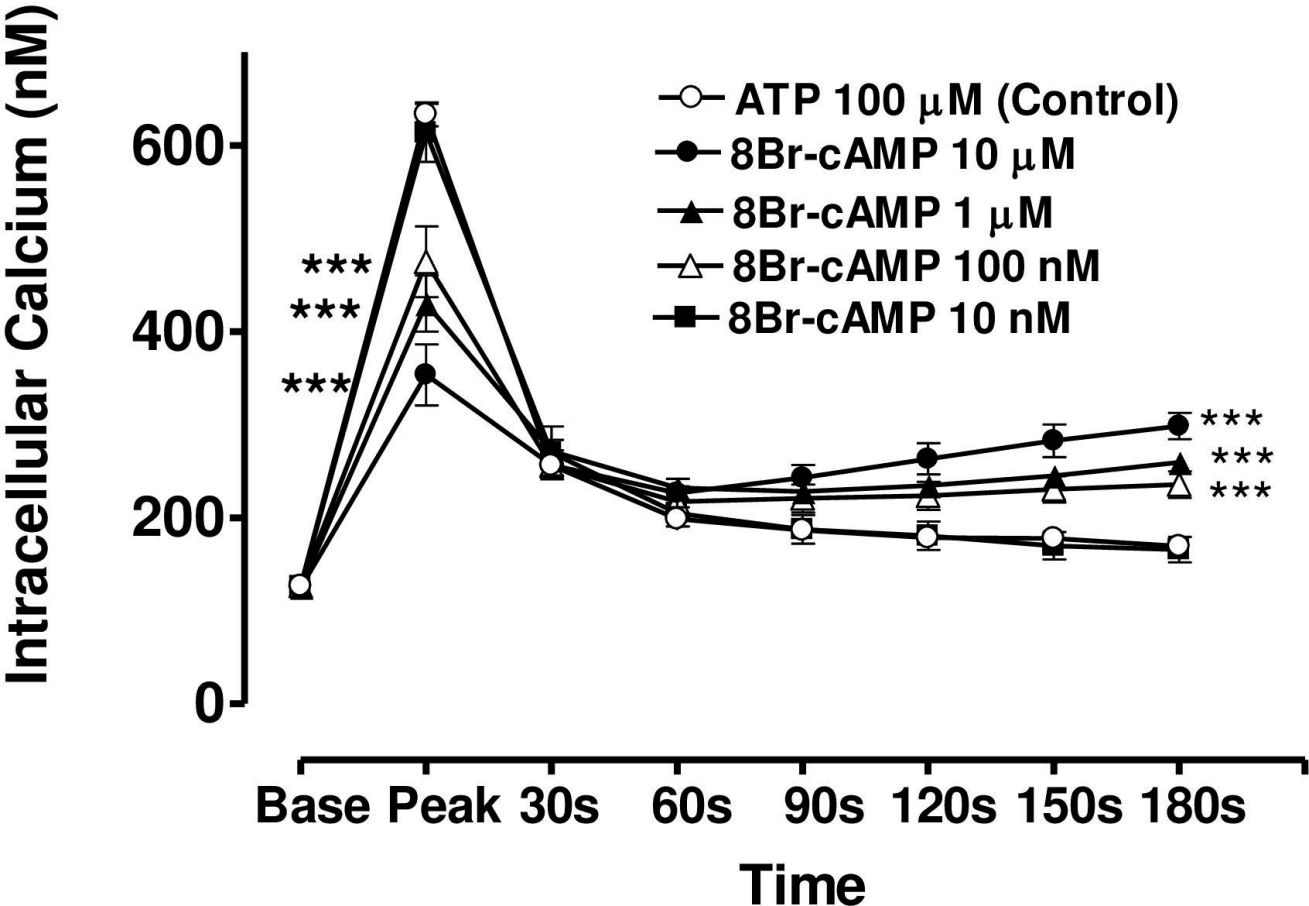
Effect of increasing concentration of 8-Br-cAMP on the Ca^2+^-mobilizing response of ATP (100μM) in bovine cultured CE, both at the peak of the Ca^2+^ transient and at selected time intervals thereafter. Cultured cells were incubated for 6 min with appropriate concentration of 8-Br-cAMP in HEPES bufererd Krebs’ solution containing 1.8mM calcium. The cells were then exposed to 100μM of ATP. Each point represents a mean±s.e.mean of 8–15 experiments. Significance of differences between incubations with or without 8-BR-cAMP; ** p < 0.01 and *** p < 0.001, Bonferroni’s multiple comparison test. Base value indicates the [Ca^2+^]_i_ prior to addition of ATP.

When the CE cells were incubated in a medium containing terbutaline, forskolin or 8-bromo cAMP, the magnitude of the peak value of the Ca2+ transient triggered by ATP was decreased, but the sustained plateau phase was increased (Figs 6, 7 and 8). In each case the effects of these three agents were concentration-dependent and the time course of the effects was very similar from one agent to the next. In the case of terbutaline (100 μM), the maximum degree of inhibition of peak height of the Ca^2+^ transient was 48.7 ± 4.5% (mean ± s.e.mean, n = 10) of control response while the degree of augmentation of the plateau phase (at 180 s) was 67.8 ± 7.3% (Fig 6). The lowest concentration of terbutaline (1 μM) which significantly depressed peak heights was also the lowest effective concentration in terms of augmenting the plateau phase (calcium entry).

In the case of forskolin (10μM), the maximum degree of inhibition of peak height of the Ca^2+^ transient was 50.1± 4.5% (mean ± s.e.mean, n = 8) of control response while the degree of augmentation of the plateau phase (at 180 s) was 63.7 ± 6.2% (Fig 7). In the case of 8-bromo cAMP (10 μM), the maximum degree of inhibition of peak height of the Ca^2+^ transient was 44.2± 5.5% (mean ± s.e.mean, n = 8) of control response while the degree of augmentation of the plateau phase (at 180 s) was 59.0 ± 10.5% (Fig 8). The lowest concentrations of forskolin and 8-bromo cAMP which significantly depressed peak heights were also the lowest effective concentrations in augmenting the plateau phase in each case.

### Effect of KT-5720 on terbutaline inhibition of the ATP-induced Ca^2+^ release

**Fig 9:**
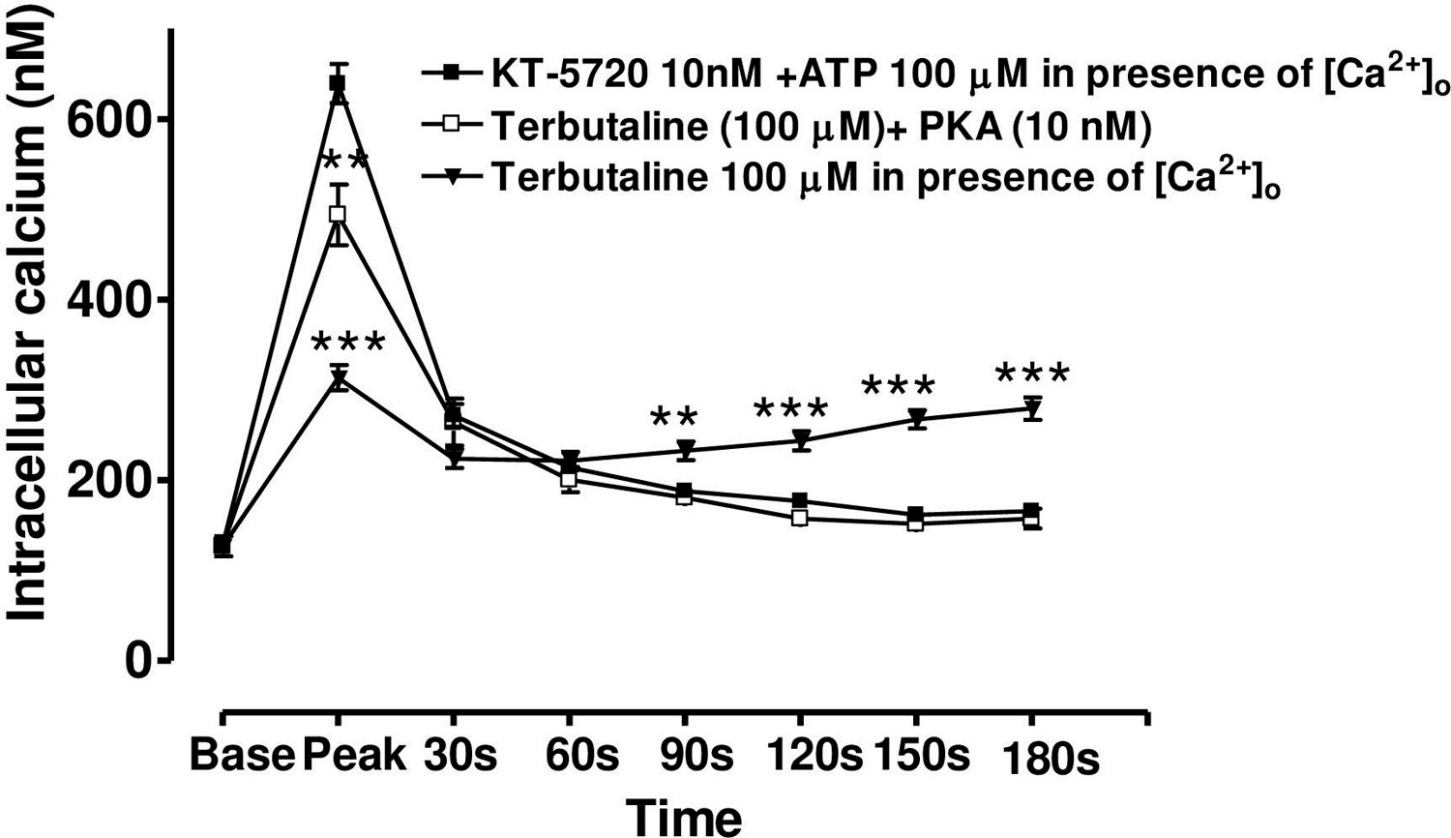
The effect of the PKA inhibitor, KT-5720 (10nM), on the terbutaline-induced inhibition of [Ca^2+^]_i_ increase by ATP. Cells were incubated with either KT-5720 (10nM), or terbutaline (100μM) alone or with KT-5720 (10nM) and terbutaline (100μM) together for 6 min before stimulating by ATP (100μM). KT-5720 largely reversed terbutaline-induced inhibition of the rapid phase of Ca2+ release by ATP. On the other hand, it completely abolished terbutaline-induced augmentation of the plateau phase of the ATP response. Each point is a mean±s.e.mean of 7–10 experiments. Significance of differences from 100μM terbutaline treated response; ** p < 0.01 and *** p < 0.001, Bonferroni’s multiple comparison test. Base value indicates the [Ca^2+^]_i_ prior to addition of ATP.

KT-5720 (10 nM) significantly (*P* < 0.001) reduced the inhibitory effect of terbutaline on the ATP-induced peak rise in [Ca2+]i from 48.7 ± 4.5% to 22.7 ± 6.2% (mean ± s.e.mean, n = 7). On the other hand, KT-5720 (10 nM) completely blocked the terbutaline’s effect on the plateau phase of the Ca^2+^ response (Fig 9).

### Effect of terbutaline on ATP-induced Ca_2+_ transients in the absence of extracellular Ca^2+^ or in presence of Ni^2+^

**Fig 10:**
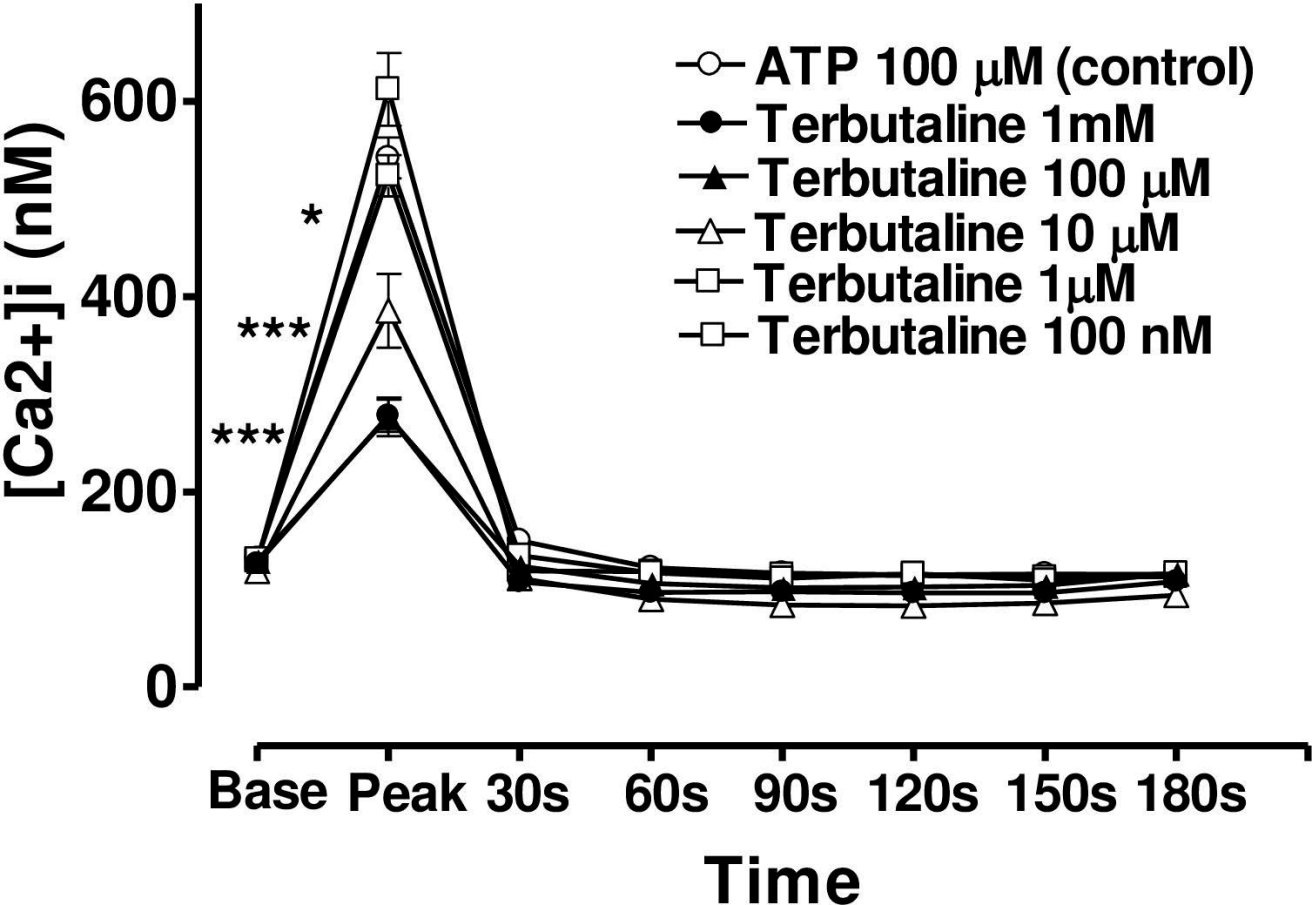
Effect of increasing concentration of terbutaline on the Ca^2+^-mobilizing response of ATP (100μM) in bovine cultured CE, in the absence of extracellular calcium. Terbutaline’s effect on both the peak Ca^2+^ transient and at selected time intervals thereafter had been shown. Cultured cells were incubated for 6 min with appropriate concentration of terbutaline in HEPES buffered Krebs’ solution containing 40mM EGTA and no calcium. The cells were then exposed to 100μM of ATP. Each point represents a mean±s.e.mean of 6–11 experiments. Significance of differences between incubations with or without terbutaline; ** p < 0.01 and *** p < 0.001, Bonferroni’s multiple comparison test. Base value indicates the [Ca^2+^]_i_ prior to addition of ATP.

**Fig 11:**
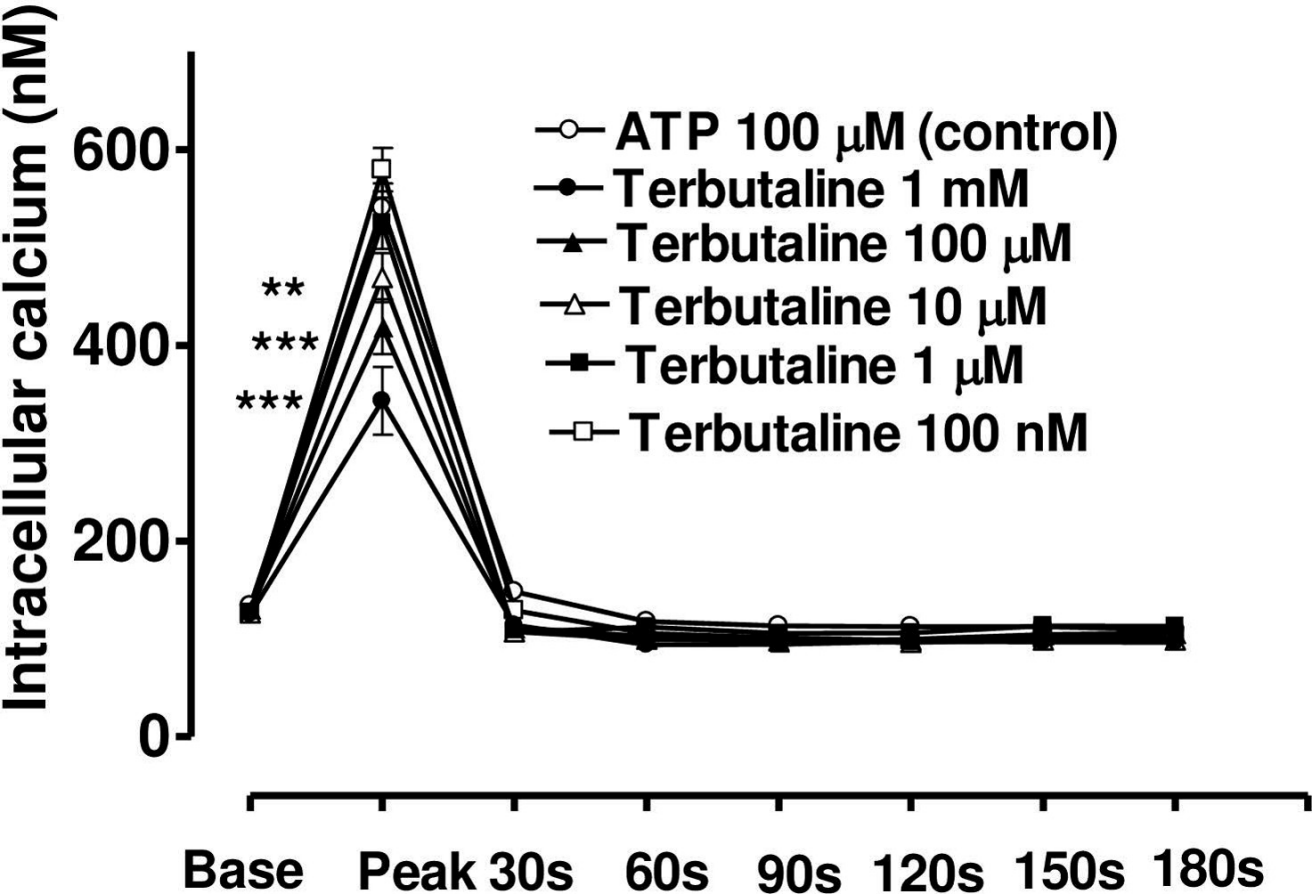
Effect of increasing concentration of terbutaline on the Ca^2+^-mobilizing response of ATP (100μM) in bovine cultured CE, in the present of extracellular calcium and NiCl_2_ (4mM). Terbutaline’s effect on both the peak Ca^2+^ transient and at selected time intervals thereafter had been shown. Cultured cells were incubated for 6 min with appropriate concentration of terbutaline in HEPES buffered Krebs’ solution containing 4mM NiCl_2_ and1.8mM calcium. The cells were then exposed to 100μM of ATP. Each point represents a mean±s.e.mean of 8–12 experiments. Significance of differences between incubations with or without terbutaline; ** p < 0.01 and *** p < 0.001, Bonferroni’s multiple comparison test. Base value indicates the [Ca^2+^]_i_ prior to addition of ATP.

When CE cells were incubated in a calcium-free medium containing EGTA (40 mM) (Fig 10), or a medium with calcium but containing NiCl_2_ (4mM) (Fig 11), the peak phase of the ATP-induced [Ca^2+^]_i_ transient was unchanged, however, the plateau phase was virtually absent. Under these conditions, terbutaline still suppressed the peak phase, but the sustained plateau phase was no longer augmented (Figs 10 and 11). This result confirms that the plateau phase is due to entry of calcium from extracellular space.

## Discussion and conclusion

The present study demonstrates that terbutaline, forskolin and 8-bromo cAMP, perfused arterially at appropriate concentrations, decrease AHF in the bovine isolated eye preparation. This confirms our previous report [12] that terbutaline lowers IOP and AHF, although our failure (in that earlier work) to demonstrate a similar effect of forskolin or 8-bromo cAMP may have been due to the nature of drug administration–by single bolus injection rather than continuous arterial perfusion. In the present work, all drugs were delivered continuously at a fixed concentration in the perfusate. A further implication of this is that, in the present work, drug concentrations perfused through the vasculature of the whole eye should closely approximate actual drug concentrations in the ciliary body and can therefore be directly compared to those applied to the CE cells in culture in the study of calcium movements.

The involvement of cAMP in the mechanism of the β-agonist effect on intraocular pressure was proposed by Neufeld [4]. We have previously summarised [12] the controversy over adrenergic effects on AH dynamics. A bolus dose of terbutaline decreased AHF in the bovine perfused eye and that the same dose also increased cAMP in the ciliary processes, although this increase was only measurable when the phosphodiesterase inhibitor, IBMX, was also present [12]. Forskolin increases cAMP synthesis by a different mechanism from terbutaline, yet it too decreases AHF, encouraging the idea that this nucleotide may mediate the effects of both drugs on AHF. Our present evidence in the bovine eye that continuous perfusion of forskolin or 8-Br-cAMP decreases AHF and that KT-5720 (a selective PKA inhibitor) inhibits the terbutaline-induced suppression of AH secretion, throws the balance of evidence in favour of the hypothesis that cAMP is the most likely mediator of the terbutaline effect on AHF in the bovine eye. The lack of an effect of KT5720 on its own also indicates that endogenous levels of cAMP in the isolated eye may be too low to exert a tonic effect on AH secretion. The exact mechanism by which cAMP reduces AH secretion in the bovine eye is not known. However, cAMP has been shown, by dual patch clamp and lucifer yellow (LY) transfer studies, to inhibit gap junctional communication between PE and nonpigmented ciliary epithelim (NPE) of bovine CE [29]. Gap junctional communication between PE and NPE is fundamentally important function in AH secretion. This is because AH is formed by ion and fluid transfer from the ciliary stroma sequentially across the PE cells, gap junctions, NPE cells and then into the posterior aqueous humor chamber of the eye [30,31]. Cyclic AMP also activate chloride channels in the PE of bovine eye [1,32]. Activation of chloride channels in the PE would favour reabsorption of chloride ions into the stroma and hence potential reduction in AH secretion. Thus, present data also supports the hypothesis that chloride ion transport across the CE is an important contributor in AH secretion [33], particularly in the bovine eye [25].

The present study demonstrates that terbutaline, forskolin and cAMP are all potent inhibitors of cytoplasmic Ca^2+^ release induced by exogenous ATP in bovine CE cells. In an earlier study we have shown that in these cells ATP mobilises [Ca^2+^]i mainly from internal stores [18]. Extracellular ATP acts on G-protein coupled cell-surface P2Y2 receptors to stimulate the membrane-bound enzyme phospholipase C, causing production of IP3, which then binds to its receptor on the endoplasmic reticulum, thus mobilising stored Ca^2+^. All three drugs raise cellular levels of cAMP and all produce a substantial and concentration-dependent inhibition of ATP-induced [Ca^2+^]i release.

In our system, the [Ca^2+^]_i_ response of CE to ATP occurs in three phases. There is evidence to suggest the rapid initial rise is due to release of intracellular stored Ca_2+_ [18] and this also occurs in other secretory cells [34]. The rapid decline which follows the peak is probably due to plasma membrane Ca-ATPase activity and sodium-calcium exchange expelling Ca_2+_ through the plasma membrane [35] and/or to reuptake of Ca_2+_ by the intracellular stores. There follows the third (plateau) phase, a much slower decline in Ca_2+_ which, in bovine CE cells, we previously reported and re-confirmed in the present study, was completely abolished in the absence of extracellular Ca_2+_ or when Ca_2+_ entry was prevented with NiCl2 [18]. Some workers describe this phase as capacitative calcium entry, since it appears to be a means of refilling the cell’s calcium stores from the extracellular pool.

In the present work, terbutaline, forskolin and cAMP all suppressed the release of stored calcium in a concentration-dependent manner. Similar inhibition of Ca^2+^ release by cAMP has been observed in many other tissues and cell types including platelets [36,37], aortic smooth muscle [38] and foetal lung epithelium [39]. It is believed that cAMP causes phosphorylation of the IP_3_ receptor [40], thereby inhibiting its ability to release Ca_2+_ as first suggested by Supattapone [41].

Terbutaline, forskolin and cAMP also had concentration-dependent effects on the plateau phase of the [Ca^2+^]_i_ record, significantly enhancing the entry of Ca_2+_. This effect confirms the hypothesis that the plateau phase is due to calcium entry since it is absent in Ca_2+_–free solutions or in the presence of NiCl_2_. Such enhancement of calcium entry by cAMP has also been reported in other tissues and cell types, including foetal lung epithelium [39], rat salivary gland [42] and HL60 promyelocytes [43]. In the present work, KT-5720 entirely abolished the enhancement of Ca_2+_ entry by terbutaline (100μM), confirming the published report in promyelocyte [43] that this effect is PKA-dependent.

It is interesting to note that as the release of stored Ca^2+^ is inhibited incrementally by increasing concentrations of terbutaline, forskolin or 8-Br-cAMP, so the stimulation of entry of extracellular Ca^2+^ increased. Hence this enhancement of calcium entry in bovine CE cells is not driven by the degree of store emptying, as has sometimes been observed [44,45] and could not be interpreted as stimulation of capacitative calcium entry. Rather it is mediated directly by cAMP itself on some other calcium entry pathways, since KT-5720 entirely blocked the terbutaline’s ability to stimulate Ca^2+^ entry. Several pathways of calcium influx, through the plasma membrane, had been proposed in response to receptor stimulation by agonists. These include receptor operated channels (ROC), G-protein operated channels (GOC), second-messenger operated channels (SMOC), or by calcium-release activated channels (CRAC). The last pathway (CRAC) has been proposed to be due to the release of calcium influx factor (CIF) in response to emptying of store, and has been defined as capacitative calcium entry [46,47]. Which of these mechanisms is affected by cAMP to enhance calcium entry in bovine CE remains to be elucidated.

The apparent enhancement of Ca^2+^ entry by cAMP-PKA signaling is entirely different from the effect of cGMP on Ca^2+^ movements in CE cells, as we observed earlier [24]. Although both nucleotides suppressed ATP-induced release of stored Ca^2+^, atriopeptin or 8-Br-cGMP suppressed Ca^2+^ entry while terbutaline, forskolin or 8-Br-cAMP enhanced the Ca^2+^ entry during the plateau phase of the response. Suppression of calcium entry by cGMP occured at a very low concentrations (lower threshold 100pM– 1.0 nM), while the enhancement of calcium entry by cAMP observed in the present study had a lower threshold concentration of 1.0μM. Furthermore, the effect of cGMP on calcium entry appeared not to be mediated by PKG [24].

How do we correlate the the biochemical response of cAMP on [Ca^2+^]_i_ with the cellular function of AH secretion by the CE? Terbutaline, forskolin and cAMP suppress the peak calcium response to a similar maximum extent as atriopeptin and cGMP [24] and also suppress AHF to a similar degree. Blockade of either protein kinase A and protein kinase G similarly leads to a partial inhibition of both the functional effect (on AH formation) and on [Ca^2+^]i release. In contrast, cGMP and cAMP have entirely different effects on calcium entry, i.e., cGMP abolishes whereas cAMP augments the Ca^2+^ entry phase (plateau). However, both cGMP and cAMP reduce AHF in the bovine eye. Hence we may conclude that the store release phase of the Ca^2+^ response is more relevant in terms of cyclic nucleotide modulation of secretory function in the CE.

In summary, our previous evidence [24] showed that atriopeptin and azide decreased AHF through generating cGMP and also interfered with ATP-induced Ca^2+^ release by a cGMP-dependent mechanism. The present data show that drugs which increase cellular cAMP levels are similarly able to suppress AH formation and interfere with ATP-induced Ca^2+^ release. This lends substantial support to the hypothesis that Ca^2+^ could be involved in the mechanism by which many drugs affect secretory function in the CE.

## Supporting information

S1 Table Effect of terbutaline on aqueous humor formation. (XLSX)

S2 Table Effect of Forskolin on aqueous humor formation. (XLSX)

S3 Table Effect of 8-Br cAMP on aqueous humor formation. (XLSX)

S4 Table Effect of KT-5720 on terbutaline-induced reduction of aqueous humor formation. (XLSX)

S5 Table (XLS)

S6 Table Effect of Terbutaline on ATP-induced ntracellular calcium mobilization. (XLSX)

S7 Table Effect of Forskolin on ATP-induced intracellular calcium mobilization. (XLSX)

S8 Table Effect of 8-Br cAMP on ATP-induced intracellular calcium mobilization. (XLSX)

S9 Table Effect of PKA inhibitor (KT-5720) on the terbutaline-induced inhibition of intracellular calciun increase by ATP. (XLSX)

S10 Table Effect of terbutaline on ATP-induced intracellular calcium mobilization in the absence of extracellular calcium. (XLSX)

S11 Table Effect of terbutaline on ATP-induced intracellular calcium mobilization in presence of extracellular Nickel chloride. (XLSX)

## Abbreviations

AH: aqueous humour
AHF: aqueous humour formation
cAMP: cyclic AMP
CE: ciliary epithelium
cGMP: cyclic GMP
DMEM: Dulbecco’s modified Eagle’s medium
DMSO: dimethyl sulfoxide
EDTA: ethylene diamine tetraacetic acid
EGTA: ethylene glycol-bis(: β-aminoethyl ether)-N,N,N’,N’-tetraacetic acid
FBS: foetal bovine serum
HEPES: (N-[2-hydroxyethylpiperazine-N’-[4-butanesulfonic acid])
IOP: intraocular pressure
NCS: newborn calf serum
NPE: Nonpigmented ciliary epithelium
PE: Pigmented ciliary epithelium
PKA: protein kinase A
PKG: protein kinase G
UTP: uridine 5´triphosphate
