# Supplementary material for: Terbutaline, forskolin and cAMP reduce secretion of aqueous humour in the isolated bovine eye
Source: PLoS One. 2020 Dec 21;15(12):e0244253. doi: 10.1371/journal.pone.0244253 (PMC7751850; doi:10.1371/journal.pone.0244253)
Supplement: Attachment — Submitted filename: Response to Reviewers-R1.docx [file pone.0244253.s012.docx]

NONE-D-2350-R1

**Response to Reviewers**

We thank the editor for the opportunity to submit a revised manuscript. We appreciate and agree with the comments made by the academic editor and the two reviewers. Point-by-point responses to each comment are listed below. Changes in the manuscript have been identified by page and line numbers and by red font in the “marked-up” copy of the manuscript.

**Response to the Editor’s comments.**

1. Ethics statement:

i). Editor wants us to include the full name of the ethics committee that approved the specific study in the Method section.

*We have now amended the ethic statement to include full name of the ethics committee: “****The use of animal tissue was approved by the University of Arizona, Institutional Animal Care and Use Committees (Protocol number: 12-395) and conformed to the ARVO Resolution for the Use of Animals in Ophthalmic and Vision Research.” (Page 5, lines 113-115).***

ii) Editor asked us to add the same statement to the “Ethics Statement” field of the submission form.

***We have now added exactly the same statement to the “Ethic Statement” field.***

2. Errors in Formatting the References.

***We have corrected the errors.***

**Reviewer 1**

**Major concerns**

1. Although the authors claim that the effects of terbutaline and other drugs functioned dose-dependently, they did not except 8-Br cAMP in Fig. 1-3.

***The reviewer is right. We have now changed the description to reflect the data (Page 8, lines 184-186).***

**Minor concerns**

1. The formats of many articles in the reference list are incorrect. For instance, "Current eye research" should be "Curr Eye Res" in #12.

***The errors in formatting the references have now been corrected.***

**Reviewer 2**

1. The reviewer wants us to change the short title to: “Cyclic AMP modulates the production of aqueous

humor.”

***We liked the suggested short title and replaced the old one (Page 1, line 14).***
