# Supplementary material for: Terbutaline, forskolin and cAMP reduce secretion of aqueous humour in the isolated bovine eye
Source: PLoS One. 2020 Dec 21;15(12):e0244253. doi: 10.1371/journal.pone.0244253 (PMC7751850; doi:10.1371/journal.pone.0244253)
Supplement: Peer review history

# Peer review: Decision Letter 0

**DOI:** 10.1371/journal.pone.0244253.r001

**Authors:** Agustín Guerrero-Hernandez

17 Nov 2020

PONE-D-20-23950

Terbutaline, forskolin and cAMP reduce secretion of aqueous humour in the isolated bovine eye.

PLOS ONE

Dear Dr. Shahidullah,

Thank you for submitting your manuscript to PLOS ONE. After careful consideration, we feel that it has merit but does not fully meet PLOS ONE’s publication criteria as it currently stands. Therefore, we invite you to submit a revised version of the manuscript that addresses the points raised during the review process.

Please submit your revised manuscript by Jan 01 2021 11:59PM. If you will need more time than this to complete your revisions, please reply to this message or contact the journal office at plosone@plos.org. When you're ready to submit your revision, log on to https://www.editorialmanager.com/pone/ and select the 'Submissions Needing Revision' folder to locate your manuscript file.

Please include the following items when submitting your revised manuscript:

- A rebuttal letter that responds to each point raised by the academic editor and reviewer(s). You should upload this letter as a separate file labeled 'Response to Reviewers'.
- A marked-up copy of your manuscript that highlights changes made to the original version. You should upload this as a separate file labeled 'Revised Manuscript with Track Changes'.
- An unmarked version of your revised paper without tracked changes. You should upload this as a separate file labeled 'Manuscript'.

If you would like to make changes to your financial disclosure, please include your updated statement in your cover letter. Guidelines for resubmitting your figure files are available below the reviewer comments at the end of this letter.

If applicable, we recommend that you deposit your laboratory protocols in protocols.io to enhance the reproducibility of your results. Protocols.io assigns your protocol its own identifier (DOI) so that it can be cited independently in the future. For instructions see: http://journals.plos.org/plosone/s/submission-guidelines#loc-laboratory-protocols

We look forward to receiving your revised manuscript.

Kind regards,

Agustín Guerrero-Hernandez

Academic Editor

PLOS ONE

Journal Requirements:

1) Thank you for including your ethics statement:

"The use of animal tissue was approved by the Institutional

114 Animal Care and Use Committees (IACUCs, Protocol number: 12-395) and conformed to the

115 ARVO Resolution for the Use of Animals in Ophthalmic and Vision Research.".

i) Please amend your current ethics statement to include the full name of the ethics committee that approved your specific study. 

ii) Once you have amended this/these statement(s) in the Methods section of the manuscript, please add the same text to the “Ethics Statement” field of the submission form (via “Edit Submission”).

For additional information about PLOS ONE submissions requirements for ethics oversight of animal work, please refer to http://journals.plos.org/plosone/s/submission-guidelines#loc-animal-research

2. One of the reviewers has made requests regarding the references. For a definitive guide regarding the formatting of references in PLOS ONE please see here: https://journals.plos.org/plosone/s/submission-guidelines#loc-references

Additional Editor Comments (if provided):

[Note: HTML markup is below. Please do not edit.]

Reviewers' comments:

Reviewer's Responses to Questions

**Comments to the Author**

1. Is the manuscript technically sound, and do the data support the conclusions?

The manuscript must describe a technically sound piece of scientific research with data that supports the conclusions. Experiments must have been conducted rigorously, with appropriate controls, replication, and sample sizes. The conclusions must be drawn appropriately based on the data presented. 

Reviewer #1: Yes

Reviewer #2: Yes

**********

2. Has the statistical analysis been performed appropriately and rigorously? 

Reviewer #1: Yes

Reviewer #2: Yes

**********

3. Have the authors made all data underlying the findings in their manuscript fully available?

The PLOS Data policy requires authors to make all data underlying the findings described in their manuscript fully available without restriction, with rare exception (please refer to the Data Availability Statement in the manuscript PDF file). The data should be provided as part of the manuscript or its supporting information, or deposited to a public repository. For example, in addition to summary statistics, the data points behind means, medians and variance measures should be available. If there are restrictions on publicly sharing data—e.g. participant privacy or use of data from a third party—those must be specified.

Reviewer #1: Yes

Reviewer #2: Yes

**********

4. Is the manuscript presented in an intelligible fashion and written in standard English?

PLOS ONE does not copyedit accepted manuscripts, so the language in submitted articles must be clear, correct, and unambiguous. Any typographical or grammatical errors should be corrected at revision, so please note any specific errors here.

Reviewer #1: Yes

Reviewer #2: Yes

**********

5. Review Comments to the Author

Please use the space provided to explain your answers to the questions above. You may also include additional comments for the author, including concerns about dual publication, research ethics, or publication ethics. (Please upload your review as an attachment if it exceeds 20,000 characters)

Reviewer #1: Shahidullah and colleagues studied the effects of terbutaline, forskolin and 8-Br-cAMP to elucidate involvement of cyclic AMP and intracellular Ca2+, [Ca2+]i, in the modulation of aqueous humour formation (AHF) in the isolated bovine eye and cultured ciliary epithelial cells. They found that AHF can be manipulated through cyclic AMP, operating via protein kinase A, which can affect [Ca2+]i homeostasis. In addition, calcium release from the intracellular store affects AHF, and interaction of [Ca2+]i with cAMP plays a role in modulating AH secretion. The text is well written, and the interpretation of data are appropriate. Although there are not much surprises in the result, the data are regid. A few concerns exist.

Major concerns:

Although the authors claim that the effects of terbutaline and other drugs functioned dose-dependently, they did not except 8-Br cAMP in Fig. 1-3.

Minor concerns:

The formats of many articles in the reference list are incorrect. For instance, "Current eye research" should be "Curr Eye Res" in #12.

Reviewer #2: I have carefully read the article by Shahidullah et al. titled Terbutaline, forskolin and cAMP reduce the secretion of aqueous humor in the isolated bovine eye.

This article introduces us to the effect that terbutaline, forskolin and cAMP have on the production of aqueous humor in perfused bovine eyes. These substances induce a decrease in its production. Although the author does not conduct innovative experiments, forskolin is known to increase intracellular levels of cAMP, but perhaps it is less known that the β2 adrenergic receptor agonist (such as terbutaline) can have an antioxidant effect and today it is known that the glaucoma has a pathogenesis related to oxidative damage. So it would have been interesting to explore this possibility.However, in this article the only thing I disagree is the short title given by the authors to this article (as it has been known for over 30 years that Ca ++ is the second messenger in all cells and which also "exploits" the cyclic AMP). Therefore, I believe there is a small contradiction, it is not the effect of cyclic AMP that decreases the production of aqueous humor, and consequently the short title of the article: "Cyclic AMP reduces the secretion of aqueous humor" it's incorrect. While I agree with the authors when they state that: cAMP plays a role in modulating AH secretion. However, the article is well conducted. well-performed experiments and statistics are correct. So the only thing I would change is the short title to: Cyclic AMP modulates the production of aqueous humor.

**********

6. PLOS authors have the option to publish the peer review history of their article (what does this mean?). If published, this will include your full peer review and any attached files.

If you choose “no”, your identity will remain anonymous but your review may still be made public.

**Do you want your identity to be public for this peer review?** For information about this choice, including consent withdrawal, please see our Privacy Policy.

Reviewer #1: No

Reviewer #2: **Yes: **Sergio Claudio Saccà

[NOTE: If reviewer comments were submitted as an attachment file, they will be attached to this email and accessible via the submission site. Please log into your account, locate the manuscript record, and check for the action link "View Attachments". If this link does not appear, there are no attachment files.]

While revising your submission, please upload your figure files to the Preflight Analysis and Conversion Engine (PACE) digital diagnostic tool, https://pacev2.apexcovantage.com/. PACE helps ensure that figures meet PLOS requirements. To use PACE, you must first register as a user. Registration is free. Then, login and navigate to the UPLOAD tab, where you will find detailed instructions on how to use the tool. If you encounter any issues or have any questions when using PACE, please email PLOS at figures@plos.org. Please note that Supporting Information files do not need this step.

# Author comment: Author response to Decision Letter 0

**DOI:** 10.1371/journal.pone.0244253.r002

24 Nov 2020

NONE-D-2350-R1

Response to Reviewers

We thank the editor for the opportunity to submit a revised manuscript. We appreciate and agree with the comments made by the academic editor and the two reviewers. Point-by-point responses to each comment are listed below. Changes in the manuscript have been identified by page and line numbers and by red font in the “marked-up” copy of the manuscript.

Response to the Editor’s comments.

1. Ethics statement: 

i). Editor wants us to include the full name of the ethics committee that approved the specific study in the Method section.

We have now amended the ethic statement to include full name of the ethics committee: “The use of animal tissue was approved by the University of Arizona, Institutional Animal Care and Use Committees (Protocol number: 12-395) and conformed to the ARVO Resolution for the Use of Animals in Ophthalmic and Vision Research.” (Page 5, lines 113-115). 

ii) Editor asked us to add the same statement to the “Ethics Statement” field of the submission form.

We have now added exactly the same statement to the “Ethic Statement” field. 

2. Errors in Formatting the References. 

We have corrected the errors.

Reviewer 1

Major concerns

1. Although the authors claim that the effects of terbutaline and other drugs functioned dose-dependently, they did not except 8-Br cAMP in Fig. 1-3.

The reviewer is right. We have now changed the description to reflect the data (Page 8, lines 184-186).

Minor concerns

1. The formats of many articles in the reference list are incorrect. For instance, "Current eye research" should be "Curr Eye Res" in #12.

The errors in formatting the references have now been corrected.

Reviewer 2

1. The reviewer wants us to change the short title to: “Cyclic AMP modulates the production of aqueous humor.”

We liked the suggested short title and replaced the old one (Page 1, line 14).

Attachment Submitted filename: Response to Reviewers-R1.docx Click here for additional data file.

# Peer review: Decision Letter 1

**DOI:** 10.1371/journal.pone.0244253.r003

**Authors:** Agustín Guerrero-Hernandez

7 Dec 2020

Terbutaline, forskolin and cAMP reduce secretion of aqueous humour in the isolated bovine eye.

PONE-D-20-23950R1

Dear Dr. Shahidullah,

We’re pleased to inform you that your manuscript has been judged scientifically suitable for publication and will be formally accepted for publication once it meets all outstanding technical requirements.

Within one week, you’ll receive an e-mail detailing the required amendments. When these have been addressed, you’ll receive a formal acceptance letter and your manuscript will be scheduled for publication.

An invoice for payment will follow shortly after the formal acceptance. To ensure an efficient process, please log into Editorial Manager at http://www.editorialmanager.com/pone/, click the 'Update My Information' link at the top of the page, and double check that your user information is up-to-date. If you have any billing related questions, please contact our Author Billing department directly at authorbilling@plos.org.

If your institution or institutions have a press office, please notify them about your upcoming paper to help maximize its impact. If they’ll be preparing press materials, please inform our press team as soon as possible -- no later than 48 hours after receiving the formal acceptance. Your manuscript will remain under strict press embargo until 2 pm Eastern Time on the date of publication. For more information, please contact onepress@plos.org.

Kind regards,

Agustín Guerrero-Hernandez

Academic Editor

PLOS ONE

Additional Editor Comments (optional):

Reviewers' comments:

Reviewer's Responses to Questions

**Comments to the Author**

1. If the authors have adequately addressed your comments raised in a previous round of review and you feel that this manuscript is now acceptable for publication, you may indicate that here to bypass the “Comments to the Author” section, enter your conflict of interest statement in the “Confidential to Editor” section, and submit your "Accept" recommendation.

Reviewer #1: (No Response)

**********

2. Is the manuscript technically sound, and do the data support the conclusions?

The manuscript must describe a technically sound piece of scientific research with data that supports the conclusions. Experiments must have been conducted rigorously, with appropriate controls, replication, and sample sizes. The conclusions must be drawn appropriately based on the data presented. 

Reviewer #1: Yes

**********

3. Has the statistical analysis been performed appropriately and rigorously? 

Reviewer #1: Yes

**********

4. Have the authors made all data underlying the findings in their manuscript fully available?

The PLOS Data policy requires authors to make all data underlying the findings described in their manuscript fully available without restriction, with rare exception (please refer to the Data Availability Statement in the manuscript PDF file). The data should be provided as part of the manuscript or its supporting information, or deposited to a public repository. For example, in addition to summary statistics, the data points behind means, medians and variance measures should be available. If there are restrictions on publicly sharing data—e.g. participant privacy or use of data from a third party—those must be specified.

Reviewer #1: Yes

**********

5. Is the manuscript presented in an intelligible fashion and written in standard English?

PLOS ONE does not copyedit accepted manuscripts, so the language in submitted articles must be clear, correct, and unambiguous. Any typographical or grammatical errors should be corrected at revision, so please note any specific errors here.

Reviewer #1: Yes

**********

6. Review Comments to the Author

Please use the space provided to explain your answers to the questions above. You may also include additional comments for the author, including concerns about dual publication, research ethics, or publication ethics. (Please upload your review as an attachment if it exceeds 20,000 characters)

Reviewer #1: (No Response)

**********

7. PLOS authors have the option to publish the peer review history of their article (what does this mean?). If published, this will include your full peer review and any attached files.

If you choose “no”, your identity will remain anonymous but your review may still be made public.

**Do you want your identity to be public for this peer review?** For information about this choice, including consent withdrawal, please see our Privacy Policy.

Reviewer #1: No

# Editor report: Acceptance letter

**DOI:** 10.1371/journal.pone.0244253.r004

**Authors:** Agustín Guerrero-Hernandez

9 Dec 2020

PONE-D-20-23950R1 

Terbutaline, forskolin and cAMP reduce secretion of aqueous humour in the isolated bovine eye. 

Dear Dr. Shahidullah:

I'm pleased to inform you that your manuscript has been deemed suitable for publication in PLOS ONE. Congratulations! Your manuscript is now with our production department. 

If your institution or institutions have a press office, please let them know about your upcoming paper now to help maximize its impact. If they'll be preparing press materials, please inform our press team within the next 48 hours. Your manuscript will remain under strict press embargo until 2 pm Eastern Time on the date of publication. For more information please contact onepress@plos.org.

If we can help with anything else, please email us at plosone@plos.org. 

Thank you for submitting your work to PLOS ONE and supporting open access. 

Kind regards, 

PLOS ONE Editorial Office Staff

on behalf of

Dr. Agustín Guerrero-Hernandez 

Academic Editor

PLOS ONE
